# Comparison of Methods for Estimating Temporal Topic Models From Primary Care Clinical Text Data: Retrospective Closed Cohort Study

**DOI:** 10.2196/40102

**Published:** 2022-12-19

**Authors:** Christopher Meaney, Michael Escobar, Therese A Stukel, Peter C Austin, Liisa Jaakkimainen

**Affiliations:** 1 Dalla Lana School of Public Health, Division of Biostatistics University of Toronto Toronto, ON Canada; 2 Department of Family and Community Medicine University of Toronto Toronto, ON Canada; 3 ICES Toronto, ON Canada; 4 Institute of Health Policy, Management and Evaluation University of Toronto Toronto, ON Canada

**Keywords:** clinical text data, temporal topic model, nonnegative matrix factorization, latent Dirichlet allocation, structural topic model, BERTopic, text mining

## Abstract

**Background:**

Health care organizations are collecting increasing volumes of clinical text data. Topic models are a class of unsupervised machine learning algorithms for discovering latent thematic patterns in these large unstructured document collections.

**Objective:**

We aimed to comparatively evaluate several methods for estimating temporal topic models using clinical notes obtained from primary care electronic medical records from Ontario, Canada.

**Methods:**

We used a retrospective closed cohort design. The study spanned from January 01, 2011, through December 31, 2015, discretized into 20 quarterly periods. Patients were included in the study if they generated at least 1 primary care clinical note in each of the 20 quarterly periods. These patients represented a unique cohort of individuals engaging in high-frequency use of the primary care system. The following temporal topic modeling algorithms were fitted to the clinical note corpus: nonnegative matrix factorization, latent Dirichlet allocation, the structural topic model, and the BERTopic model.

**Results:**

Temporal topic models consistently identified latent topical patterns in the clinical note corpus. The learned topical bases identified meaningful activities conducted by the primary health care system. Latent topics displaying near-constant temporal dynamics were consistently estimated across models (eg, pain, hypertension, diabetes, sleep, mood, anxiety, and depression). Several topics displayed predictable seasonal patterns over the study period (eg, respiratory disease and influenza immunization programs).

**Conclusions:**

Nonnegative matrix factorization, latent Dirichlet allocation, structural topic model, and BERTopic are based on different underlying statistical frameworks (eg, linear algebra and optimization, Bayesian graphical models, and neural embeddings), require tuning unique hyperparameters (optimizers, priors, etc), and have distinct computational requirements (data structures, computational hardware, etc). Despite the heterogeneity in statistical methodology, the learned latent topical summarizations and their temporal evolution over the study period were consistently estimated. Temporal topic models represent an interesting class of models for characterizing and monitoring the primary health care system.

## Introduction

### Primary Care Text Data

Electronic medical record (EMR) systems are increasingly being adopted in clinical settings across the globe [1]. As a result, health care organizations are generating, collecting, and digitally storing large volumes of routinely collected clinical information. In this study, we focused on clinical text data commonly collected in primary care EMR systems. We compared a class of unsupervised machine learning models—temporal topic models—used to characterize the latent thematic content of large document corpora and summarize latent topical dynamics over time. Temporal topic models have the potential to be applied to large unstructured clinical document collections, routinely captured in modern EMR systems, to passively characterize the primary health care system.

### Topic Models

Several methods can be used to estimate a topic model, given a document collection, and to characterize the evolution of latent topical bases over time. Latent Dirichlet allocation (LDA) [2,3] uses a Bayesian probabilistic graphical modeling framework to define a topic model. Learned topical vectors describe the affinity of a word (v=1...V) in the corpus for a particular topic (k=1...K). A latent admixing vector describes the affinity of a specific document (d=1...D) for a specific topic (k=1...K). The latent matrices in the LDA model are learned from document-word co-occurrence statistics empirically collected from the clinical note corpus. The traditional LDA model is not intended for modeling temporal document collections; however, Griffiths et al [4,5] demonstrated how simple time-stratified estimators can be used to illustrate the evolution of latent topical vectors over time. The structural topic model (STM), extends the classical LDA model, allowing either (1) the matrix of per-document topical prevalence weights or (2) the matrix of per-topic word probabilities to deterministically vary according to covariate information parameterized using a generalized linear model [6]. Several parameterizations of time can be incorporated into the generalized linear model (eg, discrete, continuous, or spline effects), allowing the STM to flexibly model the evolution of topical prevalence vectors over time. Nonnegative matrix factorization (NMF) [7-9] uses a linear algebraic framework and principles from constrained optimization for topic modeling. NMF directly estimates the parameter matrices of a topic model by factorizing an observed document term matrix (DTM) into 2 latent nonnegative matrices. One of the latent parameter matrices describes the affinity of a document (d=1...D) to a topic (k=1...K), and the other latent matrix describes the affinity of a word (v=1...V) to a topic (k=1...K). Post hoc multivariate transformations of the NMF latent parameter matrices can be used to generate estimates of topical evolution over time. Recently, neural frameworks have been developed for topic modeling, such as top2vec [10] and BERTopic [11]. The BERTopic neural topic models begin by embedding documents into a latent vector space. A finite number of clusters (k=1...K) of semantically similar documents are identified in the embedding space. For each document cluster (k), the most relevant words describing the cluster or topic are extracted using a cluster-specific term-frequency inverse-document frequency (TF-IDF) weighting technique [11].

### Study Objectives

The objective of this study was to compare the performance of several temporal topic modeling methodologies fitted to a corpus of primary care clinical notes. We compared the following temporal topic modeling methodologies: NMF, LDA, STM, and BERTopic. We examined (1) the overall matrix of per-topic word probabilities estimated over the corpus and (2) the multivariate time series structures describing the evolution of latent topical prevalence weights (k=1...K) over discrete times (t=1...T). We compared the methods using a data set of longitudinal primary care clinical notes collected over 5 years (2011-2015) in Ontario, Canada.

## Methods

### Mathematically Representing and Computationally Processing Our Clinical Text Corpus

Topic models use statistical information regarding document-word co-occurrence frequencies to learn meaningful latent variable representations from a corpus. Each document in the collection (d=1...D) is represented as a high-dimensional length-V vector (v=1...V), where each element is a count of the number of times a particular word or token (v) in an empirical vocabulary is observed in a particular document (d). We represented the collection of document-specific term-frequency vectors into a matrix X of dimension D*V, called the DTM. The DTM is a large, sparse matrix. However, the matrix is overdetermined because many of the rows (representing document-specific term-frequency vectors) and columns (representing word or token occurrence frequency over all documents in the corpus) demonstrate strong intercorrelations. Dimension-reduction techniques, such as topic models, use intercorrelated statistical semantic information to estimate meaningful thematic representations from document collections. Topic models learn (1) clusters of intercorrelated words describing the topical content of the corpus and (2) clusters of correlated documents sharing latent topical concepts.

The most challenging and subjective aspect associated with construction of the DTM involves specification of the vocabulary or dictionary (v=1...V) encoding the column space of the matrix. A priori constructed lexicons or dictionaries (of dimension V) can be used to determine the study vocabulary. Specification of appropriate domain-specific dictionaries would be tasked with subject matter experts on the research team. Alternatively, an entirely computational approach could specify a text tokenization or normalization pipeline and computationally parse the input character sequences into a finite number of tokens.

In this study, we adopted a hybrid approach to vocabulary or dictionary specification. We began by tokenizing the clinical notes on whitespace boundaries (spaces, tabs, newlines, carriage returns, etc). We normalized tokens using lower-case conversion and removed all nonalphabetic characters. We removed tokens with a character length ≤1. Finally, we sorted the list of tokens or words by decreasing occurrence frequency and manually reviewed the sorted list of tokens. Our manual review identified V=2930 distinct tokens for inclusion in our final vocabulary. The total number of tokens in the corpus was 3,003,583. The tokens chosen for inclusion in our final dictionary or vocabulary were mainly medical terms with precise semantic meanings (disease names, disease symptoms, drug names, medical procedures, medical specialties, anatomical locations, etc). We excluded stop words or tokens (ie, syntactic or functional tokens with little clinical semantic meaning). Words with low occurrence frequency were excluded for computational considerations. All text processing was conducted using R (R Foundation for Statistical Computing; version 3.6).

### Review of Methods for Temporal Topic Modeling

#### NMF Model

NMF estimates latent topical matrices using the document-word co-occurrence statistics contained in the empirical DTM. NMF factorizes the D*V dimensional DTM into 2 latent submatrices of dimensions D*K (θ) and K*V (Φ). The DTM (X) consists of nonnegative integers (ie, word frequency counts), whereas the learned matrices (θ,Φ) consist of nonnegative real values. Mathematically, the NMF objective involves learning optimal values of the latent matrices (θ,Φ) that best approximate the input data set (X ≈ θΦ), subject to the constraint that the learned matrices contain nonnegative values.



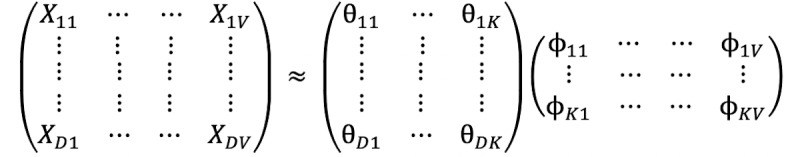



We selected a least square loss function to train the NMF model. The objective function specifies that the observed data elements are approximated in a K-dimensional bilinear form 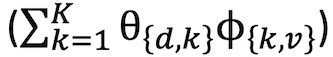. The analyst must specify the dimensions of the latent space: K (the number of topics). Seminal articles on NMF include Paatero and Tapper [7] and Lee and Seung [8,9]. Surveys of NMF and low-rank models are provided by Berry et al [12] and Udell et al [13].



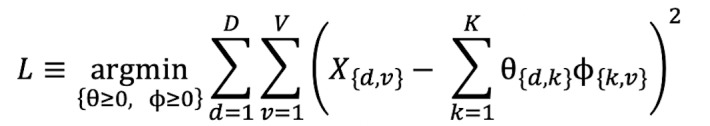



Post hoc, the row vectors constituting both θ and Φ, can be normalized by dividing by their respective row sums. The resulting normalized vectors can be interpreted as compositional or probability vectors (ie, each normalized row of θ and Φ contains nonnegative entries that sum to 1, row-wise). The row vectors of the matrix Φ encode a set of k=1...K per-topic word probabilities or proportions (estimated over a discrete set of v=1...V words in the empirical corpus vocabulary). The row vectors of the matrix θ encode a set of d=1...D per-document topic proportions (estimated over a discrete set of k=1...K latent dimensions), encoding the affinity a given document has for a particular topic.

For each document d=1...D, assume we observe a time stamp that allows us to associate each document (and latent embedding) with a T-dimensional indicator variable denoting the observation time (t=1...T). We estimated a K-dimensional multivariate mean topical prevalence vector for each design point, t=1...T. This resulted in a multivariate time series structure (a T*K dimensional matrix). Each column (k=1...K) of the matrix is a length T time series that described the evolution of a latent topical vector.

The sklearn.decomposition.NMF() function in the Python SKLearn package (version 0.24.2) was used to fit the NMF topic model.

#### LDA Model

**Figure 1:**
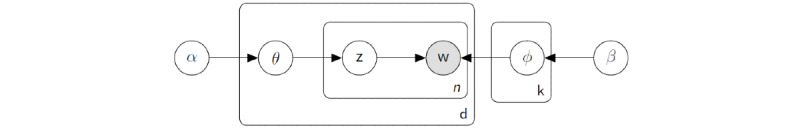
Graphical model representation of the latent Dirichlet allocation topic model.

LDA is a probabilistic topic model. Probabilistic topic models assume that a document comprises a mixture of topics. These (latent) topics represent a probability distribution over a finite vocabulary of words or tokens. Topic models can also be described as admixture models. Each document is a soft mixture of topics (k=1...K), where a topic is itself a probability distribution over words in the vocabulary (v=1...V). A graphical model describing LDA is shown in Figure 1 [2].

The LDA graphical model also describes a generative process for creating a single document in the corpus. This can be succinctly described using the following sampling notation [14,15].

To generate a document, we begin by sampling the per-topic word distributions from a Dirichlet distribution parameterized by a V dimensional prior concentration parameter (β). Topical vectors (k=1...K) are shared over the collection of documents.



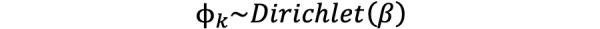



Next, for each document d=1...D in the collection, we sample the per-document topic distribution from a Dirichlet distribution parameterized according to a K-dimensional prior concentration parameter (α).



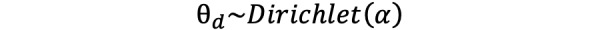



For each word in each document, we sample a topical indicator variable, z_d,n_. This variable takes an integer value between 1 and K and signifies the per-topic word distribution from which a specific word, w_d,n_, is chosen. The index n denotes the n^th^ word in a variable length document (n=1...N_d_).



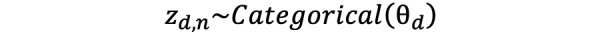



Finally, we draw a single word token, w_d,n_, from the topical distribution associated with z_d,n_. The word indicator is an element v=1...V in our empirical dictionary or vocabulary.



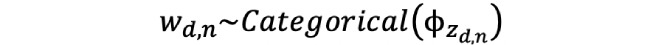



The statistical inference problem associated with probabilistic topic modeling involves inverting the sampling process and learning model-defined latent parameters given the observed text data. The latent variables indicate which words are assigned to which topical indicators (z), which documents have an affinity for which topics (θ), and which words co-occur with high likelihood under which topics (Φ). The latent parameters associated with an LDA topic model are typically estimated using Bayesian statistical machinery (Gibbs sampling [14], variational inference [2], and other methods).

A multivariate transformation of the matrix of per-document topical prevalence weights generates a multivariate time series data structure. This object is of dimension T*K, where each column k=1…K represented a univariate topical time series of length T. This series describes the evolution of latent topical vectors over our study period.

The sklearn.decomposition.LatentDirichletAllocation() function in Python SKLearn (version 0.24.2) was used to fit the LDA topic model.

#### STM Model

**Figure 2:**
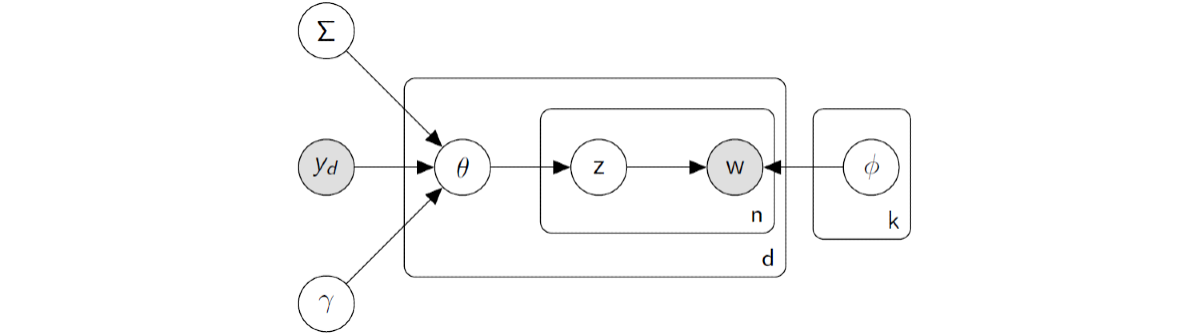
Graphical model representation of the structural topic model.

The STM is another type of probabilistic topic model. The STM extends the LDA topic model, allowing latent matrices of (1) per-document topical prevalence weights or (2) per-topic word proportions to vary according to a generalized linear model parameterization [6]. Covariate effects on the latent matrix of per-document topical prevalence weights are incorporated into the model using a logistic-normal prior distribution over per-document topical prevalence vectors, similar to the correlated topic model [16]. Covariate effects on the latent matrix of per-topic word proportions are incorporated into the model using a type of multinomial logit prior. In this study, we modeled covariate effects (in our study, discrete time effects, t=1...T) on the matrix of per-document topic prevalence weights. We did not assume that the matrix of per-topic word proportions varied according to covariates. The plate notation of STM is shown in Figure 2. Variational methods are used for posterior inference in STM [6].

To generate a document under STM, we begin by sampling the per-topic word distributions from an (intercept-only) multinomial logit model (where multinomial logit regression parameters are given sparse “gamma-lasso” prior) [6].



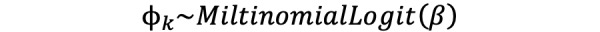



Next, we sample the per-document topic distribution from a logistic-normal distribution parameterized in terms of a mean vector and covariance matrix. γ represents a D*T dimensional design matrix encoding the time point (t=1...T) under which the document (d=1...D) was observed. The vector γ is a matrix of dimension T*K and encodes discrete time effects on each of the per-document topical prevalence weights (a length K vector for each document d=1...D). Finally, Σ is a K*K dimensional covariance matrix that encodes correlations between topical prevalence vectors (parameterized under a logistic-normal model).



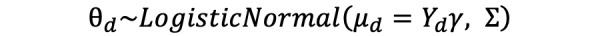



For each word (n=1...N_d_) in each document (d=1...D), we sample a topical indicator variable z_d,n_. This variable takes an integer value between 1 and K and signifies the per-topic word distribution from which a specific word, w_d,n_, is chosen. It must be noted that the upper limit N_d_ suggests that the number of words used for any given document (d) can vary.



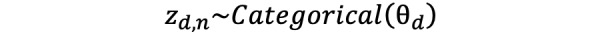



Finally, we draw a single word or token, w_d,n_, from the topical distribution associated with z_d,n_. The word indicator is an element v=1...V in our empirical dictionary or vocabulary.



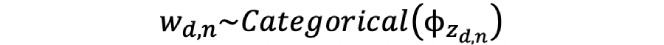



The framework for STM naturally allows for the estimation of temporal effects on topical prevalence weights. In our study, discrete time effects on topical prevalence can be interpreted using the coefficient matrix (γ) from the fitted logistic-normal model. As the temporal effects are encoded in a Bayesian regression modeling framework, we can also compute inferential measures (posterior means, highest posterior density intervals, etc). The single-stage inferential mechanism encoded in STM is a clear strength over earlier NMF and LDA models.

We used the stm() function in the STM package in R to fit the STM to our study data.

#### Neural Topic Modeling via BERTopic

Recently, researchers have developed topic models that integrate neural architectures and related techniques for model specification and learning. These neural topic models represent a different class of topic models compared with those introduced previously. Examples of recently developed neural topic models include top2vec [10] and BERTopic [11]. In this study, we focused on the BERTopic model.

BERTopic begins with embedding documents empirically observed in the study corpus into a latent embedding space. Many methods exist for embedding discrete linguistic units (words, sentences, paragraphs, documents, etc) into an embedding space. For example, words can be embedded in a vector space using word2vec [17-19], GloVe [20], FastText [21], ELMO [22], Flair [23], and transformer models [24]. Sentences and documents can be embedded using methods such as doc2vec [25], universal sentence encoders [26], and transformers [24]. The BERTopic model used in this study relies on sentence transformers [27], particularly the MPNet sentence transformer model [28]. The neural embedding model is a discrete “hyperparameter” in the BERTopic modeling pipeline. Different choices of neural embedding models are associated with their own model-specific hyperparameters (embedding dimension, context window width, model training or optimization arguments, etc).

Each document (d=1...D) is embedded in a vector space, typically of a few hundred dimensions. The uniform manifold approximation and projection (UMAP) algorithm [29] was used as a further nonlinear dimension-reduction technique to assist in the visualization and clustering of document vectors. Clustering was accomplished in the UMAP-reduced space using the hierarchical density-based spatial clustering algorithm of applications with noise (HDBSCAN) [30].

Clusters (k=1...K) of semantically related documents were identified. Scores over words v=1...V in the vocabulary were computed using cluster-specific TF-IDF weights. If a cluster consisted of semantically focused documents, and hence words, we expect to observe coherent and meaningful words identified via TF-IDF scoring. The proportion of documents assigned to each cluster during a specific period (t=1...T) can be used to generate a T*K dimensional multivariate time series structure, depicting the evolution of latent topic over our study period.

We fitted the BERTopic model using default hyperparameter settings. The BERTopic pipeline requires (1) specification of a document embedding algorithm (in our case, the MPNet sentence transformer model [28]), (2) the UMAP nonlinear dimension-reduction algorithm, (3) the HDBSCAN algorithm for cluster identification, and (4) cluster-specific TF-IDF scoring. The individual components of the pipeline could involve substantive hyperparameter optimization. In this study, we used the default model hyperparameter settings.

We used the Python package bertopic to fit BERTopic models.

### Statistical Methods for Corpus Description and Evaluation of Learned Temporal Topic Models

We used simple counts and percentages to describe the characteristics of our study sample. We described the number of unique patients and number of unique clinical notes. Each patient in our sample was a “high-user” of the primary care system, in the sense they generated at least one encounter/note for each of the twenty quarterly time periods between 2011-2015. We described the distribution of the number of notes per patients. We described demographic characteristics of the sample (age/sex distributions).

When fitting the NMF, LDA, and STM models, we constructed a DTM whose row dimension corresponded to the number of unique patients in the sample (ie, 1727 unique patients) multiplied by the number of distinct time periods (*t*=20; 1727×20=34,540). Each term-frequency vector observed in the DTM was length V (V=2930), and an individual element counted the number of times a given word was observed for a given patient in each quarterly period. Across the DTM, we counted the total number of words and the number of unique words. We described the counts and percentages of the top 25 most prevalent words in our clinical note corpus. We also described the sparsity of the DTM.

For each of the NMF, LDA, STM, and BERTopic models, we constructed a K*T dimensional multivariate time series matrix (this is the transpose of the T*K data structure described earlier). Each row corresponds to a latent topic vector and each column corresponds to a specific quarterly time period. A row vector is a length T time series describing the evolution of a latent topical vector across the study periods. Each column corresponds to a distribution over topics at a particular period (ie, described which topics are most important at a given period). For each row k=1...K, we report the top 5 words loading most strongly on a given topic. The cluster of words was semantically correlated and described the essence of the latent topical vector. A heatmap was used to visualize this high-dimensional multivariate time series structure; and we hierarchically clustered the rows of the matrix using a Euclidean distance metric and Ward agglomeration method (a dendrogram was used to visualize the cluster structure of the topical series).

The topical structure of each of the NMF, LDA, STM, and BERTopic model fits was described in terms of the top 5 words loading most strongly on each of the k=1...K latent topics. In other words, the topical structure of each model can be described in terms of a “bag” of 250 words or tokens. We investigated the topical diversity of the model fits. Topical diversity was calculated in terms of the number of unique words in the bag of 250 total words. Furthermore, we investigated the top 5 most frequently occurring words in the “bag” describing each model fit. The redundantly occurring words in the topical summaries provided a rough approximation of the semantic concepts that the models repeatedly identified as important.

We investigated several measures of topical coherence for the NMF, LDA, STM, and BERTopic models. We considered the “UMASS,” “UCI,” and normalized pointwise mutual information (“NPMI”) metrics described in the surveys of Roder et al [31] and Rosner et al [32]. These metrics assessed the internal consistency of the collection of word clusters describing the topical structure of the NMF, LDA, STM, and BERTopic models. The theoretical minima or maxima of each coherence measure varies; however, larger values indicate models that generated more coherent topical characterizations. Mathematical details related to the calculation of the aforementioned topical coherence metrics are provided later and further outlined in the studies by Roder et al [31] and Rosner et al [32]. In all the equations used, we assumed that a topical vector is described in terms of its top-L most probable words or tokens; {*w_i_,w_j_*} represented distinct words from the top-L set, ε is a small positive constant to avoid potential numerical issues in computation; and δ is a weighting term (used in the normalized NPMI estimates, compared with the unnormalized pointwise mutual information estimates used in the UCI coherence measure).



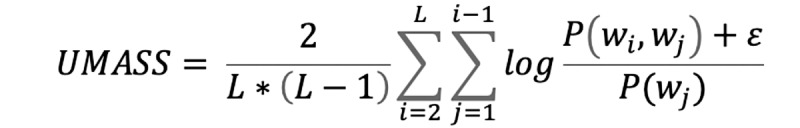





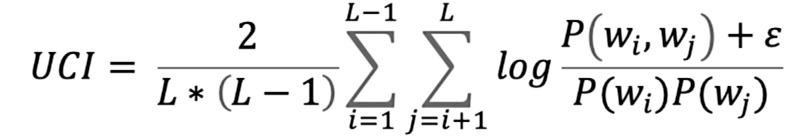





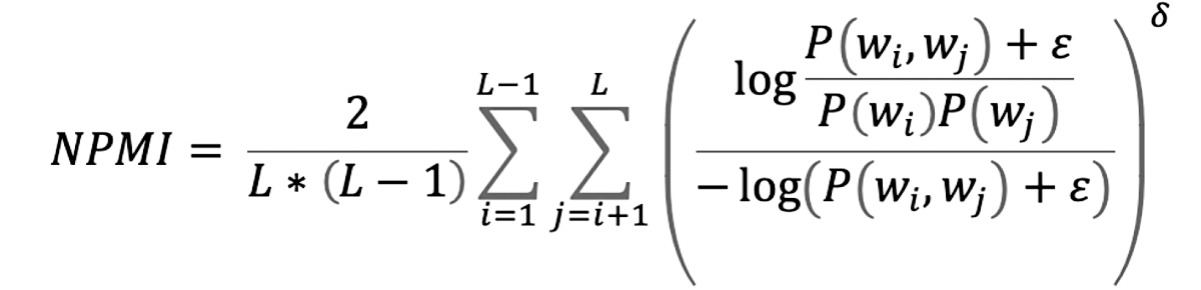



We used a set-based measure of concordance, the Jaccard coefficient, to assess similarities or differences in the topical structure describing the NMF, LDA, STM, and BERTopic models. Each model was described in terms of a “bag” of 250 words or tokens (ie, k=50 topics, described in terms of their top 5 most probable words); consider 2 models generating bags of words or tokens, b_0_ and b_1_. The Jaccard coefficient is defined as the cardinality of the intersection of b_0_ and b_1_ divided by the cardinality of the union of b_0_ and b_1_. In mathematical notation, the Jaccard coefficient is expressed as follows:



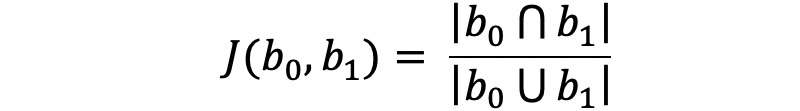



Finally, we described the wall time (in seconds or minutes) required to fit each of the NMF, LDA, STM, and BERTopic models. We also discussed the computational issues associated with hyperparameter tuning of each of the models.

### Study Design, Setting, Data Sources, and Inclusion or Exclusion Criteria

This study used a retrospective closed cohort design. Clinical notes were obtained from primary care EMR systems geographically distributed across Ontario, Canada. We included all clinical notes written by the patient’s primary care provider between January 01, 2011, and December 31, 2015. We discretized time into quarterly strata (January-March; April-June; July-September; and October-December). Patients were excluded if they did not have at least one clinical note in each of the 20 quarterly strata over the study period. Hence, the selected sample of patients reflects a unique set of individuals who frequently engaged with the primary health care system.

## Results

### Description of Corpus and Study Sample

Our document collection contained 160,478 clinical notes from 1727 patients. The 1727 patients received primary care services from 1066 unique primary care physicians at 40 unique primary care clinics (geographically distributed across Ontario, Canada). The median age of the patients was 68 (IQR 55-80) years and ranged from 20 to 103 years (age statistics were calculated using study baseline as a reference date, January 1, 2011). Female patients were observed more frequently than male patients (1157/1727, 67% vs 570/1727, 33%). Table 1 describes the characteristics of the study sample (in terms of both note-level and patient-level units of analysis).

The initial note-level DTM had dimensions of 160,478 rows (one row for each clinical note in the corpus) by 2930 columns (one column for each unique word or token in the corpus). The corpus comprised 3,003,583 tokens. The DTM was >99% sparse (ie, it contained almost all zero elements). We also constructed a patient-quarter–level DTM by aggregating notes observed on the same patient within a quarter. This DTM had dimensions of 1727×20=34,540 rows by 2930 columns and was >98% sparse. The top 25 most frequently occurring words in the analytic corpus are listed in Table 2.

**Table 1:** Descriptive statistics for study sample, at note-level and patient-level unit of analysis.

Characteristic		Unique notes (n=160,478), n (%)	Unique patients (n=1727), n (%)
**Age (years)**			
	20-40	9713 (6.1)	107 (6.1)
	40-65	63,588 (39.6)	675 (39.1)
	65-85	63,839 (39.8)	704 (40.8)
	>85	23,338 (14.5)	241 (14)
**Sex**			
	Male	51,530 (32.1)	570 (33)
	Female	108,948 (67.9)	1157 (67)
**Year**			
	2011	28,012 (17.5)	—^a^
	2012	31,220 (19.5)	—
	2013	33,676 (21)	—
	2014	33,756 (21)	—
	2015	33,814 (21)	—

^a^Not applicable.

**Table 2:** Top 25 most frequently occurring tokens or words in the final analytic primary care clinical note corpora (N=3,003,583).

Token or word	Occurrence frequency, n (%)
pain	88,132 (2.93)
mg	65,612 (2.18)
inr	52,970 (1.76)
bp	50,751 (1.69)
back	43,556 (1.45)
dose	29,861 (0.99)
feels	24,736 (0.82)
rx	23,211 (0.77)
chest	22,256 (0.74)
meds	20,914 (0.7)
referral	19,409 (0.65)
work	19,398 (0.65)
wt	19,322 (0.64)
feeling	17,415 (0.58)
blood	16,121 (0.54)
symptoms	15,905 (0.53)
prn	15,706 (0.52)
urine	14,633 (0.49)
bw	13,779 (0.46)
lab	13,543 (0.45)
clear	13,271 (0.44)
knee	12,677 (0.42)
pharmacy	12,503 (0.42)
sleep	12,331 (0.41)
prescription	11,945 (0.4)

### Comparing Temporal Topic Models Estimated With NMF, LDA, STM, and BERTopic Models

We comparatively evaluated inferences obtained from fitting the NMF, LDA, STM, and BERTopic models to our primary care clinical note corpus. For each model, we varied the number of topics (K={25,40,45,50,55,60,75}) and observed similar inferences at various levels of the model complexity parameter (K). When K was too small, distinct semantic topics tended to be grouped together, whereas when K was too large, semantically similar topics tended to be split into arbitrary clusters (resulting in an overclustering effect). Using human judgment evaluation, we determined that a model complexity of K=50 topics balanced a parsimonious, while simultaneously expressive, characterization of the clinical document corpus. For each of the NMF, LDA, STM, and BERTopic models, we reported the results assuming K=50 latent topics.

**Figure 3:**
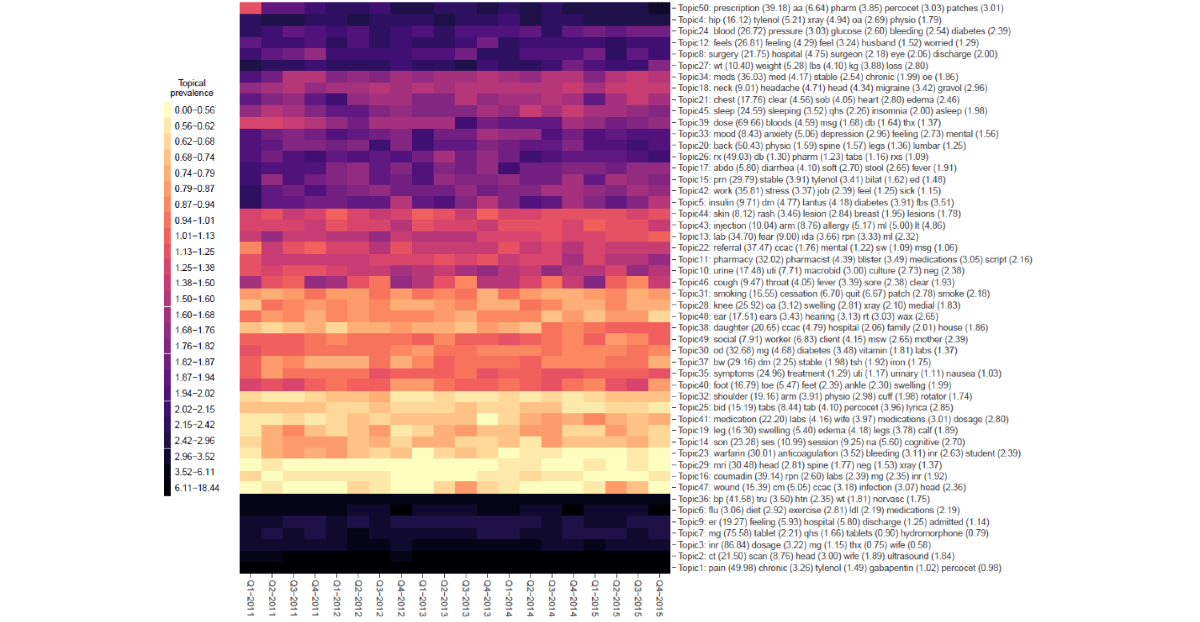
A heat map of the multivariate time series structure associated with the nonnegative matrix factorization temporal topic model.

**Figure 4:**
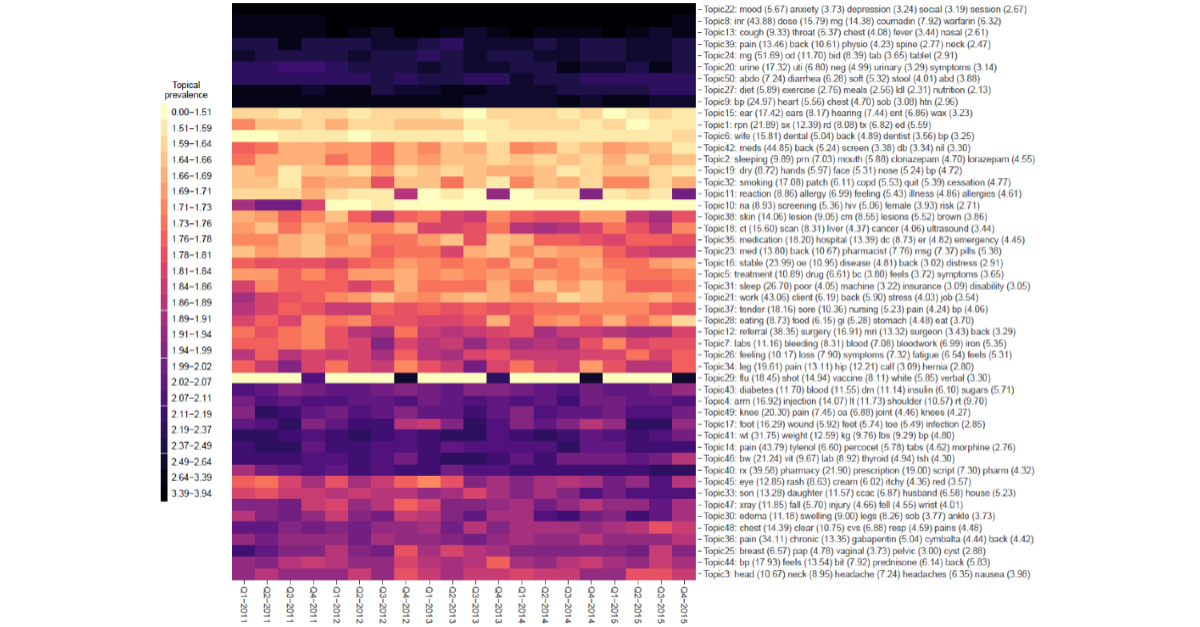
A heat map of the multivariate time series structure associated with the latent Dirichlet allocation temporal topic model.

**Figure 5:**
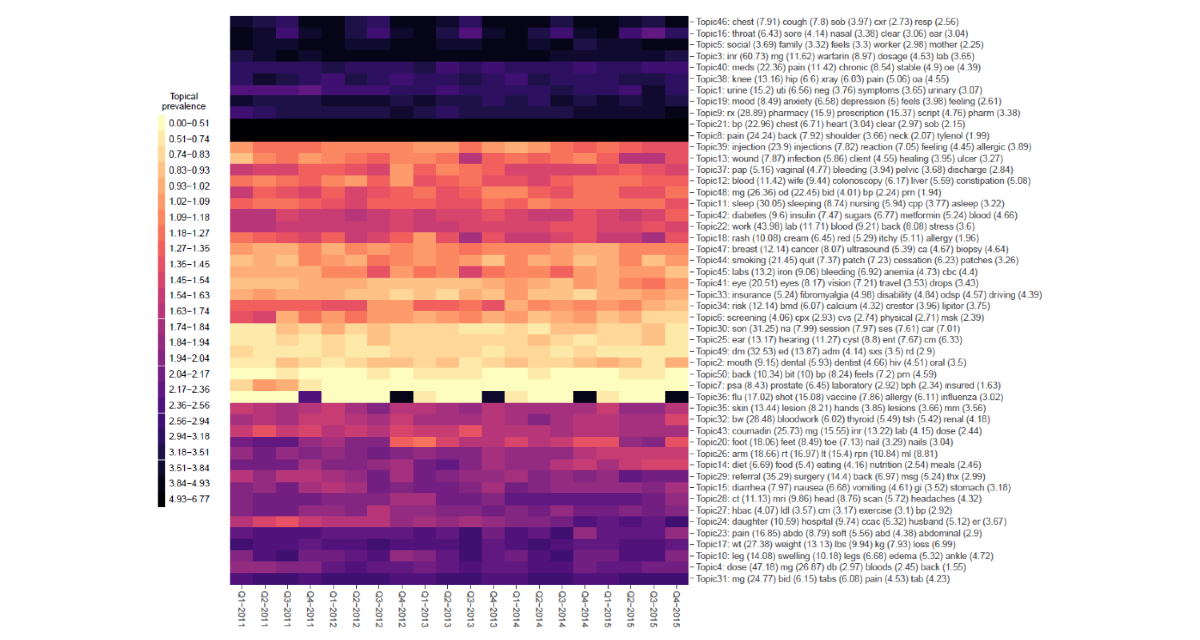
A heat map of the multivariate time series structure associated with the structural topic model temporal topic model.

**Figure 6:**
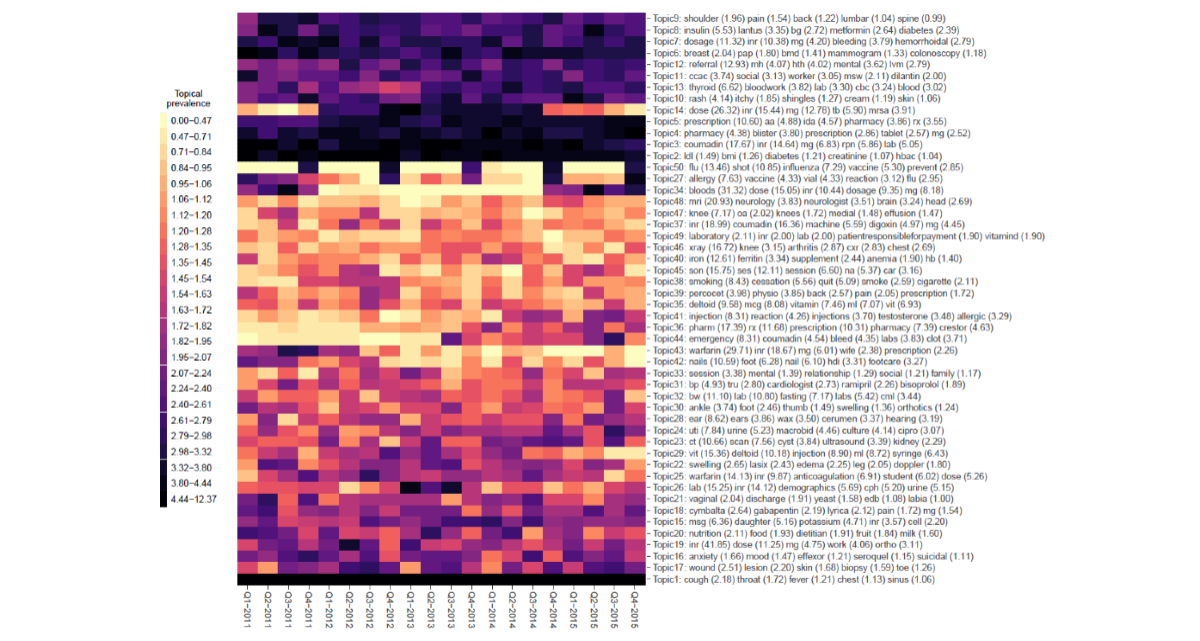
A heat map of the multivariate time series structure associated with the BERTopic temporal topic model.

A summary of the distribution of words over the k=1...50 latent topics (for each of the 4 models under comparison) is given in Figures 3-6, respectively. The y-axis in each figure lists the top 5 words loading most strongly on a given topic. For NMF, LDA, and STM, we reported topical prevalence weights associated with each word or token (which is approximately the probability of observing the word or token under a given latent topic). For the BERTopic model, we reported normalized cluster-specific TF-IDF scores associated with words under topics (which can be interpreted similarly to the outputs of the NMF, LDA, and STM models). The x-axis of these plots represents t=1...20 quarterly periods. A column in the plot represents a topical prevalence distribution over latent topics at a given time point. A row in the plot illustrates the evolution of a latent topic over the study period.

Each of the 4 latent temporal topic models learned a meaningful representation of the primary care clinical notes corpus. In the following paragraphs, we discuss (1) topics consistently estimated across models that demonstrated constant trends in topical prevalence across quarterly periods and (2) topics consistently estimated across quarterly periods that demonstrated interesting seasonal patterns.

Each of the fitted models consistently identified the following latent primary care topical constructs (and these topics show constant patterns across quarterly periods): sleep (NMF=Topic−45; LDA=Topic-2 or Topic-31; STM=Topic-11; BERTopic=not applicable); mental health, for example, mood, anxiety, and depression, (NMF=Topic-33; LDA=Topic-22; STM=Topic-19; BERTopic=Topic-16); pain (NMF=Topic-1; LDA=Topic-39, Topic-36, Topic-14, Topic-49, Topic-34, or Topic-37; STM=Topic-8; BERTopic=Topic-9 or Topic-39); blood pressure control and monitoring (NMF=Topic-36; LDA=Topic-9; STM=Topic-21; BERTopic=Topic-31); respiratory disease, for example, cough, throat, chest, fever, etc (NMF=Topic-46; LDA=Topic-13; STM=Topic-46; BERTopic=Topic-1), smoking (NMF=Topic-31; LDA=Topic-32; STM=Topic-44; BERTopic=Topic-38); diabetes, for example, blood, sugar, insulin, fbs, etc (NMF=Topic-5; LDA=Topic-43; STM=Topic-42; BERTopic=Topic-8); pharmaceutical prescription management (NMF=Topic-26; LDA=Topic-40; STM=Topic-9; BERTopic=Topic-36 or Topic-5); and annual influenza vaccination programs (NMF=Topic-6; LDA=Topic-29; STM=Topic-36; BERTopic=Topic-50). These thematic areas represented archetypical patients, conditions, or roles encountered in the primary health care system. The consistent extraction of latent themes (represented as semantically correlated word clusters) suggests that each model can leverage information regarding word-context co-occurrence to learn meaningful patterns from a large unstructured clinical document corpus.

**Figure 7:**
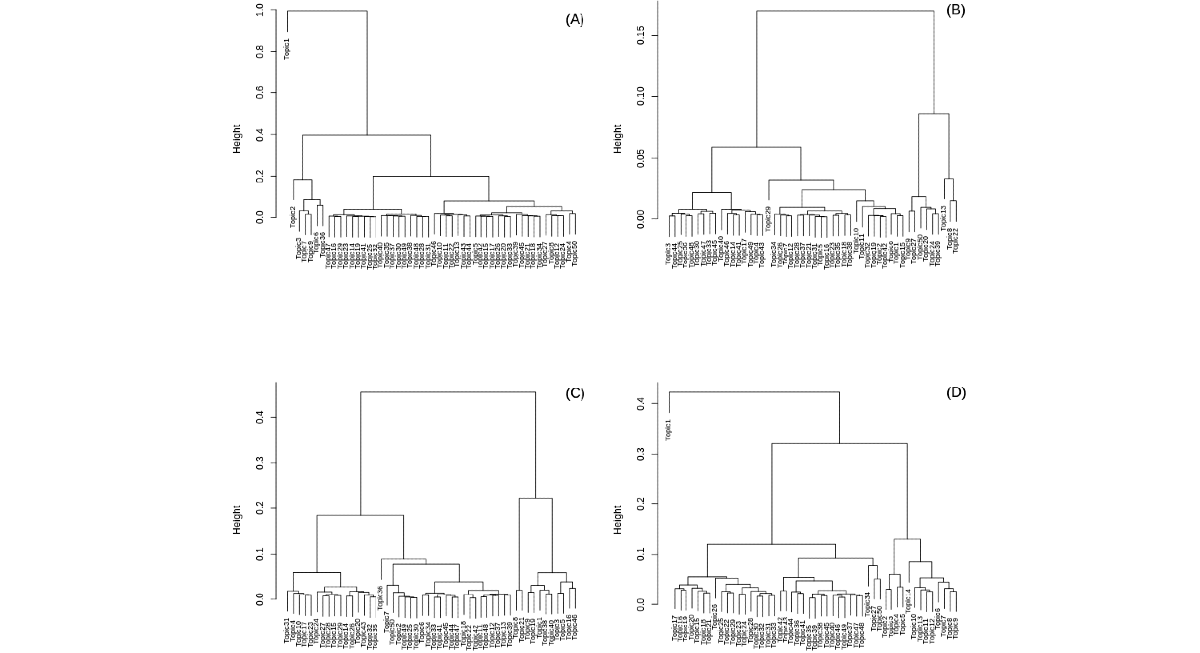
Dendrograms displaying the clustering structure of the latent multivariate time series objects learned from nonnegative matrix factorization model (A), latent Dirichlet allocation model (B), structural topic model (C) and BERTopic model (D).

Figures 3-6 illustrate 4 different temporal topic model multivariate time series structures. For a given plot, the x-axis represents time (t=1...20 quarterly periods from 2011-2015), and the y-axis represents a topical vector (k=1...50). The intensity of color in the cell (t,k) indicates the extent to which an encounter at time (t) is related to a latent topic (k). Topical labels are exchangeable and clustered along the y-axis, according to the similarity of the topical time series (a dendrogram describing the similarity or differences across topical clusters is illustrated in Figure 7). Figure 3-6 represent different multivariate time series structures estimated with NMF (Figure 3), LDA (Figure 4), STM (Figure 5), and BERTopic (Figure 6).

**Figure 8:**
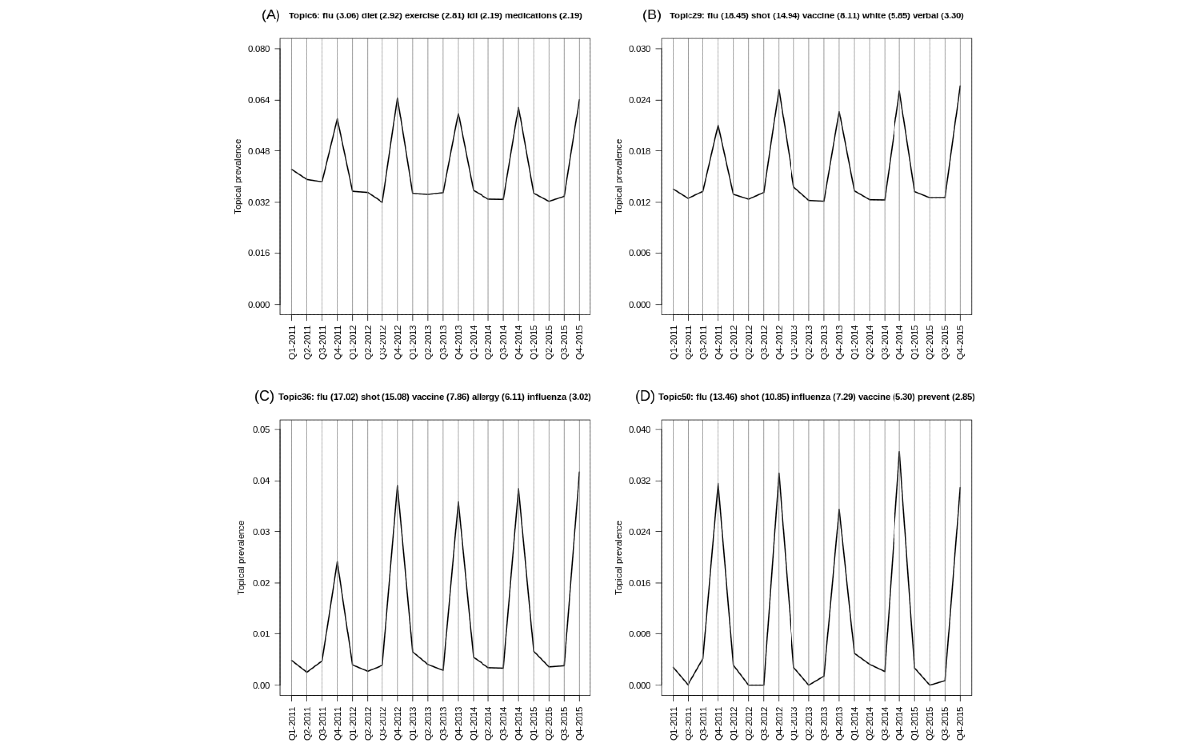
Descriptive time series plots characterizing the seasonal evolution of annual influenza program topic, as estimated by nonnegative matrix factorization model (A), latent Dirichlet allocation model (B), structural topic model (C) and BERTopic-models (D).

**Figure 9:**
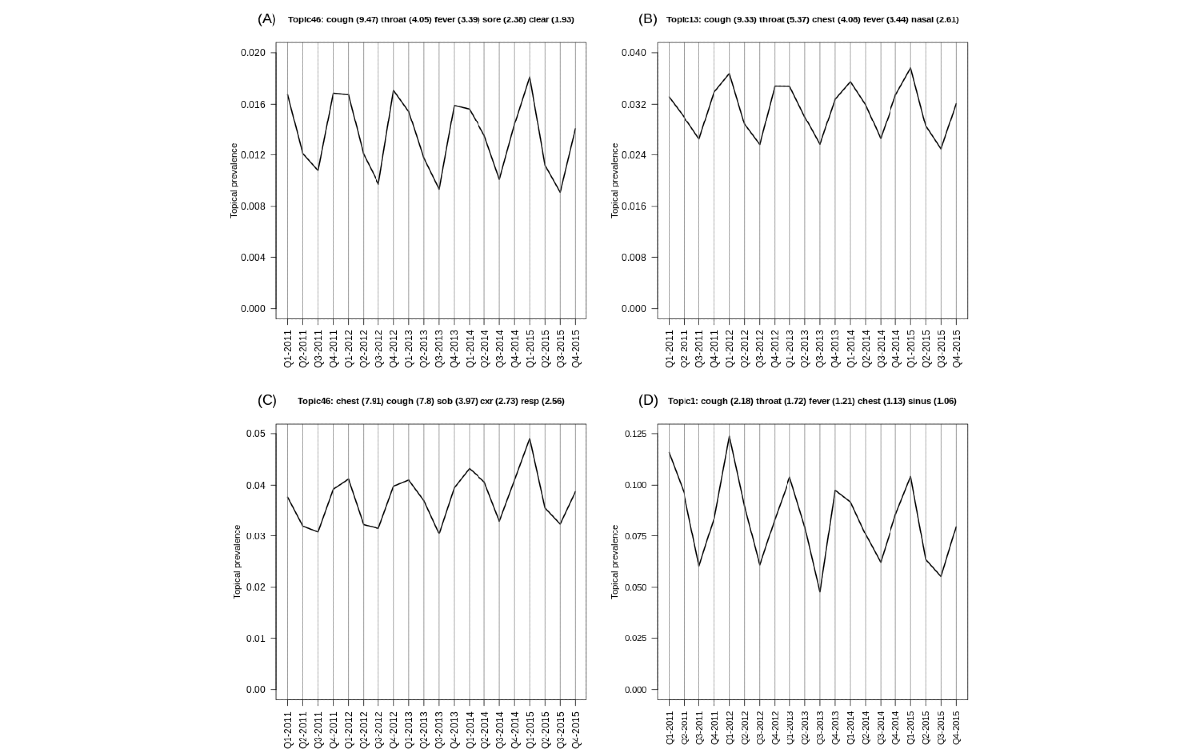
Descriptive time series plots characterizing the seasonal evolution of the respiratory disease topic, as estimated by nonnegative matrix factorization model (A), latent Dirichlet allocation model (B), structural topic model (C) and BERTopic-models (D).

For certain learned topics, seasonal harmonic patterns were stably estimated over the study period. For example, the annual influenza vaccination program consistently occurred in the fall or winter months of the study (NMF=Topic-6; LDA=Topic-29; STM=Topic-36; BERTopic=Topic-50). Similarly, annual spikes in respiratory diseases (cough, cold, influenza, etc) are identified as achieving peaks in the winter months and lows in the summer months (NMF=Topic-46; LDA=Topic-13; STM=Topic-46; BERTopic=Topic-1). These findings are illustrated in Figures 3-6; however, we also present individual time series plots of these topics in Figures 8 and 9, so the reader can better appreciate the ability of the different temporal topical models to extract consistent seasonal patterns from the primary care clinical document corpus. Findings regarding consistent seasonal variation in primary care roles over time have strong face validity and are corroborated by complementary data sources (eg, administrative data). Furthermore, the consistency by which these patterns are extracted from our large clinical document collection helps build trust in the opportunity to use word-context co-occurrence statistics (and topic models) to characterize and monitor primary care practices and systems.

### Post Hoc Internal Evaluation of Fitted Temporal Topic Models

When investigating the top-ranked words associated with per-word topic distributions in Figures 3-6 we note that each model can describe the corpus using a “bag” of up to 250 unique words (K=50 topics multiplied by top 5 words being presented for each latent topical representation). The number of unique words—also known as the topic diversity—observed in NMF, LDA, STM, and BERTopic model fits was 76.4% (191/250), 88.4% (221/250), 87.6% (219/250), and 77.2% (193/250), respectively. The top 5 most frequently recurring words or tokens describing the topical structure of each of the NMF, LDA, STM, and BERTopic models are listed in Table 3. Recurring words for LDA and STM are similar, suggesting that primary care issues related to back pain (and other musculoskeletal pain) are important, as are issues related to hypertension and feelings (eg, mood disorders). Conversely, the BERTopic model seems to prioritize primary care issues related to prescription drugs and laboratory ordering or management.

We explored the semantic coherence of NMF, LDA, STM, and BERTopic models using the following metrics: “UMASS,” “UCI,” and “NPMI” (Table 4) [31,32]. Larger coherence metrics indicated increasingly internally consistent latent topical characterizations. The “UMASS” metric favored the STM model, whereas, the “UCI” and “NPMI” metrics favored the BERTopic model.

To investigate the differences and similarities in the fitted topic model, we used the Jaccard coefficient (Table 5). Using the Jaccard measure of concordance, the Bayesian models (LDA or STM) were identified as resulting in the most similar fit. The BERTopic model generated the most distinct topical representation compared with the other models.

The time required to train each model was reported. For NMF, LDA, and STM models, we used a single central processing unit (although Python SKLearn implementations of decomposition models can be parallelized). For the BERTopic model, we used a single graphics processing unit for embedding documents and a single central processing unit for dimensionality reduction (UMAP) and clustering (HDBSCAN). Under these settings, the time required to fit the NMF, LDA, STM, and BERTopic models was 237 seconds, 67 seconds, 879 seconds (14.7 minutes), and 2624 seconds (43.7 minutes), respectively. The computational requirements of the BERTopic model exceeded those of the other models, particularly the highly optimized NMF or LDA implementations in Python SKLearn.

**Table 3:** The most frequently occurring tokens observed in each of the bags of 250 words describing the topical structure of latent Dirichlet allocation (LDA), nonnegative matrix factorization (NMF), structural topic model (STM) and BERTopic model fits (and their occurrence counts in the bag).

Word or token	Topic model			
	NMF (n)	LDA (n)	STM (n)	BERTopic (n)
Word or token-1	head (4)	back (9)	back (5)	inr (11)
Word or token-2	mg (4)	bp (6)	mg (5)	mg (9)
Word or token-3	ccac (3)	pain (6)	pain (5)	lab (5)
Word or token-4	diabetes (3)	chest (3)	bp (4)	prescription (5)
Word or token-5	feeling (3)	feels (3)	feels (3)	dose (4)

**Table 4:** Topical coherence measures (“UMASS,” “UCI,” and normalized pointwise mutual information [“NPMI”]) estimated on each of the nonnegative matrix factorization (NMF), latent Dirichlet allocation (LDA), structural topic model (STM) and BERTopic models.

Topical coherence measure	Topic model			
	NMF	LDA	STM	BERTopic
UMASS	−2.522	−2.488	−2.372	−2.591
UCI	1.220	0.987	1.192	1.405
NPMI	0.183	0.149	0.190	0.230

**Table 5:** Jaccard coefficient metrics of set-based concordance between fitted topic models: nonnegative matrix factorization (NMF), latent Dirichlet allocation (LDA), structural topic model (STM), and BERTopic.

	NMF	LDA	STM	BERTopic
NMF	—^a^	—	—	—
LDA	0.526	—	—	—
STM	0.491	0.577	—	—
BERTopic	0.343	0.286	0.329	—

^a^Not applicable.

## Discussion

### Principal Findings

In this study, we compared several distinct methodologies (ie, NMF, LDA, STM, and BERTopic) to estimate temporal topic models from a large collection of primary care clinical notes. Despite differences in the underlying statistical methodology, models often converged on a consistent latent characterization of the corpus. Furthermore, the temporal evolution of latent topics was reliably extracted from each of the NMF, LDA, STM, and BERTopic models.

Clinically, our data set represented high-users of the primary care system. Many of the latent topics emerging from this analysis are consistent with a high-user archetype, for example, family counseling or social work, mood disorders, anxiety or depression, chronic pain, arthritis and musculoskeletal disorders, neurological conditions, cardiovascular disease and hypertension, diabetes, cancer screening (breast, cervical, colorectal, and prostate), laboratory requisitions and blood work, diagnostic imaging, and pharmaceutical or prescription management. Topic models also identified numerous acute health conditions as important latent themes, such as cough, cold and other respiratory infections, urinary tract infections, skin conditions, and wound care. NMF, LDA, STM, and BERTopic models each consistently captured (1) annual primary care influenza programs and (2) seasonal respiratory conditions, demonstrating predictable seasonal variation. Findings regarding primary care use patterns, extracted solely from clinical text data, were largely corroborated by provincial reporting based on structured administrative data [33].

We observed that disparate statistical methodologies for estimating temporal topic models generated a concordant or consistent latent representation. We interpreted this to mean that as the signal-to-noise ratio increases in a given clinical text data set, the subtle choice of statistical methodology seems to matter less, and any of these methods would extract a meaningful latent representation of the primary care corpus. For smaller corpora, where word-document co-occurrence statistics are less certain, this hypothesis may not hold.

Furthermore, subtle or nuanced differences in model representations emerged, which may lead analysts to favor specific modeling strategies in particular settings. For example, consider Figure 8 for the annual influenza vaccination program. Models such as NMF and LDA are purely unsupervised and do not consider external covariate information when formulating the model objective function. For NMF or LDA models we noticed that the “grand mean” topical prevalence over time centers at approximately 2% (ie, 1/50 topics). Conversely, an STM intentionally incorporates covariate information in the Bayesian graphical models’ prior structure, and we observed that for STM, the lows for annual influenza topic are much closer to 0%, whereas the fall or winter peaks are more pronounced. The BERTopic model does not intentionally incorporate covariate information into its objective function(s) either; however, it adopts a more “local averaging” principle to estimate topical distributions over time and, as such, demonstrated similar seasonal harmonic patterns as STM in the context of the annual influenza program. Similar patterns can be observed in Figure 9 for seasonal respiratory diseases. This suggests that different topic models may perform more or less optimally in certain scientific settings (ie, may be dependent on the research question, available data, and how these foundational aspects of a study interplay with model choice). A priori, should the analyst or researcher expect topical prevalence to vary about select observable covariates, it may make sense to adopt a more flexible model that can adequately incorporate this anticipated behavior. If there is no a priori rationale to believe that topical prevalence varies as a function of covariates (eg, time in this study), then the choice of model may become less relevant, as all models may perform similarly well.

Because of the different statistical principles associated with each temporal topic modeling methodology, each method is associated with its own strengths and weaknesses. We have elaborated on the methodological and computational issues associated with each class of models.

First, NMF is the most mature and seemingly parsimonious methodology for topic modeling. NMF is strongly rooted in linear algebraic principles and is fundamentally based on the constrained optimization of a simple least squares objective function. Vanilla NMF is a well-studied statistical methodology and many efficient computational routines exist for estimating NMF models. NMF is flexible and can be readily extended. Possible model extensions can be viewed as discrete tunable hyperparameters in the model fitting process. Berry et al [12] and Cichocki et al [34] discussed distinct algorithmic techniques for estimating the latent parameters of an NMF model, such as gradient descent, multiplicative updates, and alternating nonnegative least squares. The choice of algorithm can be conceived as a discrete tunable hyperparameter. Furthermore, analysts are often confronted with the choice of whether to regularize the latent parameter matrices [35]. Ridge, lasso, and elastic net regularization are commonly encountered, although more complex regularization can be used to encourage latent representations with smoothness, minimal volume, and other characteristics. Furthermore, many researchers have attempted to introduce coherent generalizations of NMF and related techniques [13]. For example, generalized low-rank models that flexibly incorporate different loss functions, functional forms, weighting of data points, and regularization have been discussed by Udell et al [13].

LDA and STM are Bayesian topic models. LDA was developed as a fully Bayesian extension of existing linear algebraic-based (eg, latent semantic analysis) and maximum likelihood-based (eg, probabilistic latent semantic indexing) techniques for topic modeling [2]. LDA has been extended in various ways, illustrating the flexibility of Bayesian probabilistic graphical models. For example, STM is a direct extension of LDA, which allows latent parameter matrices to vary as a function of observed covariates [6]. Efficient computational fitting routines have been developed for LDA, and STM to a certain extent. Analysts face several decisions when fitting LDA and STM models to empirical data sets, including Bayesian inferential or computational methods (eg, Gibbs sampling vs variational inference) and prior distribution specifications.

BERTopic represents the most novel approach to topic modeling [11]. The BERTopic model is a pipeline: (1) deep neural networks (eg, sentence transformer models) embed documents in a vector space; (2) nonlinear dimension reduction is applied to latent document vectors (UMAP); (3) document clusters are identified (HDBSCAN); and (4) representative topics (collections of semantically correlated words) are extracted from document clusters using a cluster-specific TF-IDF scoring method. A disadvantage of the BERTopic pipeline is related to computational requirements. For large corpora, a graphics processing unit is required to learn document embeddings within a reasonable time. In our study, we randomly down-sampled our data set (3/8 documents were included, whereas 5/8 documents were excluded), even with a graphics processing unit. That said, the BERTopic model’s strength is related to its modularity. We observed that the BERTopic model generates meaningfully coherent topics, and as neural embedding methods continue to evolve, we anticipate that state-of-the-art document embedding techniques can be dropped into this pipeline.

### Limitations and Future Work

We attempted to be transparent with respect to how our final vocabulary of words or tokens was selected and accordingly the DTMs were constructed for this study. Different computational pipelines could have been used to preprocess our clinical text corpus. For instance, we could have used different strategies for tokenization, lemmatization, stemming, stop-word removal, and frequency-based word or token removal. Different text preprocessing pipelines would ultimately lead to different DTM structures (with different vocabularies). Further research is needed to better understand the implications of these text preprocessing decisions on downstream study inferences.

Each topic model considered in this study requires specification of hyperparameters that govern the aspects of model fitting. Fitting these topic models is computationally intensive for large input data sets. We focused mainly on the stability and robustness of inferences with respect to model complexity (K), a common hyperparameter across all models. We did not explore the stability of the inferences across other model-specific hyperparameters.

We did not consider all possible methods for estimating temporal topic models in this study. Bespoke NMF and LDA variants exist that are applicable for estimating temporal topic models. Sequential NMF [36] and dynamic LDA [37] are 2 extensions which are relevant for estimating temporal topic models. Tensor factorization models such as the canonical polyadic decomposition or Tucker decomposition, which factorize a D*V*T tensor into meaningful latent parameter matrices, may also be applicable [34,38]. Additional surveys related to topic modeling are provided in the studies by Churchill and Singh [39], Zhao et al [40], and Boyd-Graber et al [41].

These works have led us to consider several possible ways of extending different topic modeling frameworks, including Bayesian NMF with document-level covariates (similar to the STM extension of LDA), neural matrix factorization with (nontemporal) covariates, LDA or STM extensions that allow per-document topical prevalence weights to vary according to a flexible generalized linear mixed model or multilevel model (for modeling dependencies introduced because of the complex design or sampling mechanism by which documents are created), and computational methods for improving statistical inference (eg, interval estimation and hypothesis testing) when engaging with temporal topic models (eg, resampling methods, bootstrap, and multiple outputation).

### Conclusions

In this study, we compared several statistical techniques for estimating temporal topic models from primary care clinical text data. Different temporal topic models have unique strengths and weaknesses owing to their underlying statistical properties. Nonetheless, each model consistently estimated a latent variable representation of a primary care document collection, which meaningfully characterized high-use primary care patients and their longitudinal interactions with the primary health care system. As the adoption of EMRs increases and health care organizations amass increasingly large volumes of clinical text data, temporal topic models may offer a mechanism for leveraging unstructured clinical text data for characterization and monitoring of primary care practices and systems.

## Abbreviations

DTM: document term matrix
EMR: electronic medical record
HDBSCAN: hierarchical density-based spatial clustering algorithm of applications with noise
LDA: latent Dirichlet allocation
NMF: nonnegative matrix factorization
NPMI: normalized pointwise mutual information
STM: structural topic model
TF-IDF: term-frequency inverse-document frequency
UMAP: uniform manifold approximation and projection
